# *N*-(5-Chloro-2-nitro­phen­yl)-2,2-di­methyl­propanamide

**DOI:** 10.1107/S1600536812027730

**Published:** 2012-08-25

**Authors:** Feng Zhang, Zheng Fang, Bao-Hua Zou, Guo Kai

**Affiliations:** a School of Pharmaceutical Sciences, Nanjing University of Technology, Puzhu South Road No. 30 Nanjing, Nanjing 210009, People’s Republic of China; b College of Life Science and Pharmaceutical Engineering, Nanjing University of Technology, Puzhu South Road No. 30 Nanjing, Nanjing 210009, People’s Republic of China

## Abstract

In the crystal structure of the title compound, C_11_H_13_ClN_2_O_3_, mol­ecules are linked through C—H⋯O hydrogen bonds.

## Related literature

For background to the biologically active mol­ecule ezetimibe [systematic name: (3*R*,4*S*)-1-(4-fluoro­phen­yl)-3-[(3*S*)-3-(4-fluoro­phen­yl)-3-hy­droxy­prop­yl]-4-(4-hy­droxy­phen­yl)azetidin-2-one, see: Rosenblum *et al.* (1998 ▶). For the preparation of the title compound, a derivative of an inter­mediate in the synthesis of ezetimibe, see: Wang *et al.* (2009) ▶. For a related structure, see: Zhu *et al.* (2007 ▶).
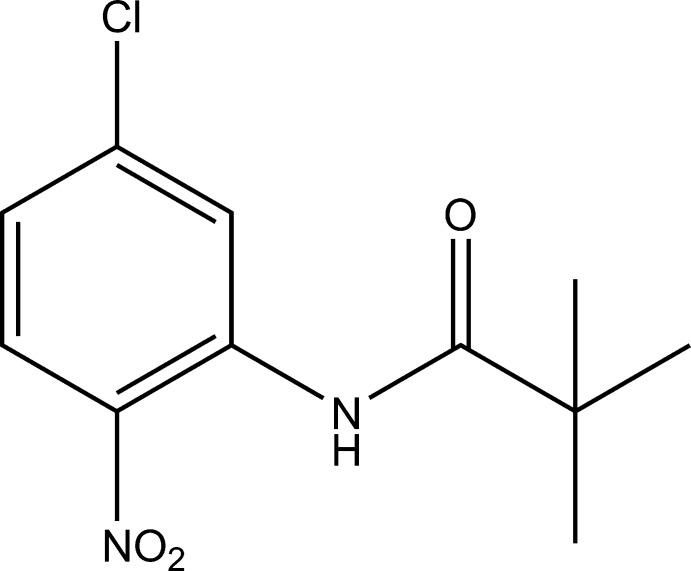


## Experimental

### 

#### Crystal data

C_11_H_12_ClN_2_O_3_
*M* *_r_* = 255.68
Orthorhombic, 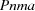

*a* = 10.401 (2) Å
*b* = 7.0280 (14) Å
*c* = 17.106 (3) Å
*V* = 1250.4 (4) Å^3^
*Z* = 4
Mo *K*α radiation
μ = 0.30 mm^−1^
*T* = 293 K
0.30 × 0.20 × 0.10 mm

#### Data collection

Enraf–Nonius CAD-4 diffractometer
Absorption correction: ψ scan (North *et al.*, 1968 ▶) *T*_min_ = 0.915, *T*_max_ = 0.970
2432 measured reflections
1244 independent reflections
643 reflections with *I* > 2σ(*I*)
*R*_int_ = 0.069
3 standard reflections every 200 reflections intensity decay: 1%

#### Refinement

*R*[*F* ^2^ > 2σ(*F* ^2^)] = 0.064
*wR*(*F* ^2^) = 0.179
*S* = 1.00
1244 reflections
94 parameters
H-atom parameters constrained
Δρ_max_ = 0.24 e Å^−3^
Δρ_min_ = −0.22 e Å^−3^

### 

Data collection: *CAD-4 Software* (Enraf–Nonius, 1989 ▶); cell refinement: *CAD-4 Software*; data reduction: *XCAD4* (Harms & Wocadlo, 1995 ▶); program(s) used to solve structure: *SHELXS97* (Sheldrick, 2008 ▶); program(s) used to refine structure: *SHELXL97* (Sheldrick, 2008 ▶); molecular graphics: *SHELXTL* (Sheldrick, 2008 ▶); software used to prepare material for publication: *PLATON* (Spek, 2009 ▶).

## Supplementary Material

Crystal structure: contains datablock(s) global, I. DOI: 10.1107/S1600536812027730/zj2083sup1.cif

Structure factors: contains datablock(s) I. DOI: 10.1107/S1600536812027730/zj2083Isup2.hkl

Supplementary material file. DOI: 10.1107/S1600536812027730/zj2083Isup3.cml

Additional supplementary materials:  crystallographic information; 3D view; checkCIF report

## Figures and Tables

**Table 1:** Hydrogen-bond geometry (Å, °)

*D*—H⋯*A*	*D*—H	H⋯*A*	*D*⋯*A*	*D*—H⋯*A*
C10—H10*A*⋯O3^i^	0.96	2.35	3.294 (8)	167

Symmetry code: (i) 
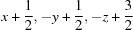
.

**Fig. 1.**
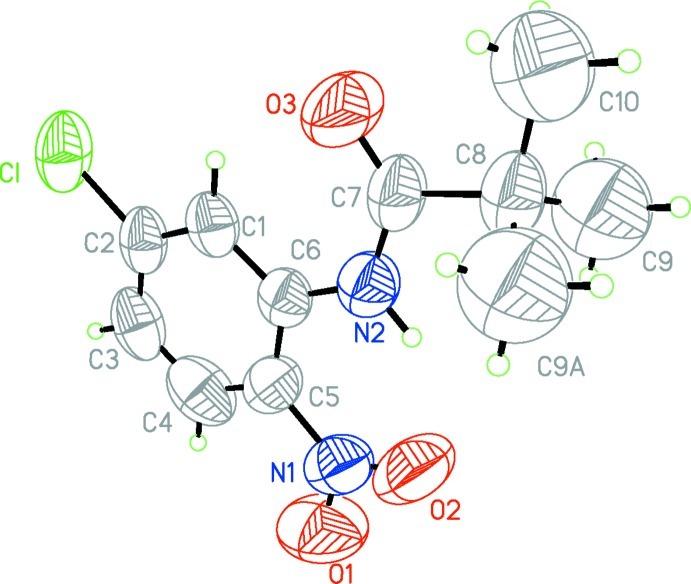
The molecular structure of the title compound with the atom numbering scheme. Displacement ellipsoids are drawn at the 50% probability level. H atoms are presented as small spheres of arbitrary radius.

**Fig. 2.**
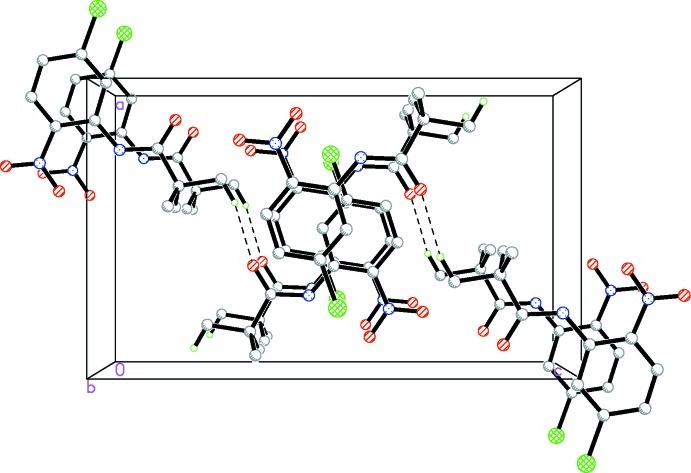
A view of the unit cell packing of the title compound.

## supplementary crystallographic information

### Comment

Ezetimibe is a biologically active molecule and reasearch has shown it to have the useful property of inhibiting the absorption of cholesterol from the intestine (Rosenblum *et al.*, 1998) As part of our studies into the synthesis of Ezetimibe, the title compound *N*-(5-chloro-2-nitrophenyl)-2,2-dimethylpropanamide, (I), which is one of the derivates of a intermediate, is synthesized (Wang *et al.*, 2009). In this paper we report the crystal structure of the title compound.

In the crystal structure,*C*—H···O hydrogen bonds interactions (Table 1) link the molecules (Fig. 2), in which they may be effective in the stabilization of the structure. It's just formed by the accumulation of Molecules.

### Experimental

5-chloro-2-nitroaniline (C_6_H_5_ClN_2_O_2_, 20.64 g, 0.12 mol) in CH_2_Cl_2_(40 ml) was added 4-dimethylaminopyridine (C_7_H_10_N_2_, 1.2 g, 0.01 mol) and Et_3_N (42.3 ml, 0.31 mol) and cooled the reaction to 273 K. A solution of pivaloyl chloride (C_5_H_9_ClO, 14.4 g, 0.12 mol) in CH_2_Cl_2_ (150 ml) was added dropwise over 1 h and the mixture was heated to reflux. After 12 h, H_2_O and H_2_SO_4_ (2 N, 75 ml) were added, separated the layers andwashed the organic layer sequentially with NaOH (10%), NaCl (satd) and water. Dried the organic layer over MgSO_4_ and concentrated to obtain solid product as pure yellow solid. (Wang *et al.*, 2009). Crystals of (I) suitable for X-ray diffraction were obtained by slow evaporation of an ethanol solution.

### Refinement

All H atoms were positioned geometrically and refined using a riding model, with C—H = 0.93 and 0.97 Å, for aryl and methylene H-atoms, respectively. The *U*_iso_(H) were allowed at 1.2*U*_eq_(C).

### Crystal data

**Table:**

C_11_H_12_ClN_2_O_3_	*F*(000) = 532
*M**_r_* = 255.68	*D*_x_ = 1.358 Mg m^−^^3^
Orthorhombic, *P**n**m**a*	Mo *K*α radiation, λ = 0.71073 Å
Hall symbol: -P 2ac 2n	Cell parameters from 25 reflections
*a* = 10.401 (2) Å	θ = 9–12°
*b* = 7.0280 (14) Å	µ = 0.30 mm^−^^1^
*c* = 17.106 (3) Å	*T* = 293 K
*V* = 1250.4 (4) Å^3^	Block, colorless
*Z* = 4	0.30 × 0.20 × 0.10 mm

### Data collection

**Table:**

Enraf–Nonius CAD-4 diffractometer	643 reflections with *I* > 2σ(*I*)
Radiation source: fine-focus sealed tube	*R*_int_ = 0.069
Graphite monochromator	θ_max_ = 25.4°, θ_min_ = 2.3°
ω/2θ scans	*h* = 0→12
Absorption correction: ψ scan (North *et al.*, 1968)	*k* = −8→0
*T*_min_ = 0.915, *T*_max_ = 0.970	*l* = −20→20
2432 measured reflections	3 standard reflections every 200 reflections
1244 independent reflections	intensity decay: 1%

### Refinement

**Table:**

Refinement on *F*^2^	Primary atom site location: structure-invariant direct methods
Least-squares matrix: full	Secondary atom site location: difference Fourier map
*R*[*F*^2^ > 2σ(*F*^2^)] = 0.064	Hydrogen site location: inferred from neighbouring sites
*wR*(*F*^2^) = 0.179	H-atom parameters constrained
*S* = 1.00	*w* = 1/[σ^2^(*F*_o_^2^) + (0.084*P*)^2^] where *P* = (*F*_o_^2^ + 2*F*_c_^2^)/3
1244 reflections	(Δ/σ)_max_ < 0.001
94 parameters	Δρ_max_ = 0.24 e Å^−^^3^
0 restraints	Δρ_min_ = −0.22 e Å^−^^3^

### Special details

**Table:**

Geometry. All e.s.d.'s (except the e.s.d. in the dihedral angle between two l.s. planes) are estimated using the full covariance matrix. The cell e.s.d.'s are taken into account individually in the estimation of e.s.d.'s in distances, angles and torsion angles; correlations between e.s.d.'s in cell parameters are only used when they are defined by crystal symmetry. An approximate (isotropic) treatment of cell e.s.d.'s is used for estimating e.s.d.'s involving l.s. planes.
Refinement. Refinement of *F*^2^ against ALL reflections. The weighted *R*-factor *wR* and goodness of fit *S* are based on *F*^2^, conventional *R*-factors *R* are based on *F*, with *F* set to zero for negative *F*^2^. The threshold expression of *F*^2^ > σ(*F*^2^) is used only for calculating *R*-factors(gt) *etc*. and is not relevant to the choice of reflections for refinement. *R*-factors based on *F*^2^ are statistically about twice as large as those based on *F*, and *R*- factors based on ALL data will be even larger.

### Fractional atomic coordinates and isotropic or equivalent isotropic displacement parameters (Å^2^)

**Table:**

	*x*	*y*	*z*	*U*_iso_*/*U*_eq_	Occ. (<1)
Cl	0.24447 (9)	0.2500	0.50682 (10)	0.0967 (6)	
N1	0.7748 (5)	0.2500	0.3855 (3)	0.0880 (14)	
C1	0.4982 (4)	0.2500	0.5237 (3)	0.0623 (11)	
H1A	0.4794	0.2500	0.5769	0.075*	
O1	0.7853 (5)	0.2500	0.3151 (3)	0.143 (2)	
N2	0.7290 (3)	0.2500	0.5525 (2)	0.0706 (11)	
H2A	0.8046	0.2500	0.5321	0.085*	
O2	0.8711 (4)	0.2500	0.4258 (3)	0.1202 (15)	
C2	0.4005 (4)	0.2500	0.4726 (3)	0.0664 (12)	
O3	0.6267 (3)	0.2500	0.6688 (2)	0.1179 (16)	
C3	0.4218 (5)	0.2500	0.3921 (3)	0.0824 (15)	
H3A	0.3541	0.2500	0.3565	0.099*	
C4	0.5469 (7)	0.2500	0.3681 (3)	0.0964 (18)	
H4A	0.5640	0.2500	0.3147	0.116*	
C5	0.6479 (4)	0.2500	0.4186 (3)	0.0732 (13)	
C6	0.6275 (4)	0.2500	0.5004 (3)	0.0585 (11)	
C7	0.7243 (4)	0.2500	0.6321 (3)	0.0720 (14)	
C8	0.8531 (4)	0.2500	0.6754 (3)	0.0802 (15)	
C9	0.9244 (4)	0.4286 (7)	0.6607 (3)	0.156	
H9A	0.9438	0.4385	0.6060	0.233*	
H9B	1.0029	0.4280	0.6902	0.233*	
H9C	0.8726	0.5351	0.6763	0.233*	
C10	0.8297 (7)	0.2500	0.7644 (4)	0.148 (3)	
H10A	0.9098	0.2500	0.7922	0.222*	
H10B	0.7814	0.3615	0.7780	0.222*	0.50

### Atomic displacement parameters (Å^2^)

**Table:**

	*U*^11^	*U*^22^	*U*^33^	*U*^12^	*U*^13^	*U*^23^
Cl	0.0576 (8)	0.0846 (10)	0.1478 (14)	0.000	−0.0208 (7)	0.000
N1	0.091 (3)	0.067 (3)	0.106 (4)	0.000	0.027 (3)	0.000
C1	0.066 (3)	0.043 (2)	0.078 (3)	0.000	−0.013 (3)	0.000
O1	0.168 (4)	0.172 (5)	0.089 (3)	0.000	0.042 (3)	0.000
N2	0.062 (2)	0.075 (3)	0.074 (3)	0.000	−0.001 (2)	0.000
O2	0.087 (3)	0.146 (4)	0.128 (4)	0.000	0.040 (3)	0.000
C2	0.053 (2)	0.045 (2)	0.101 (3)	0.000	−0.015 (2)	0.000
O3	0.060 (2)	0.215 (5)	0.078 (2)	0.000	−0.0037 (18)	0.000
C3	0.088 (4)	0.058 (3)	0.101 (4)	0.000	−0.038 (3)	0.000
C4	0.138 (5)	0.072 (4)	0.079 (4)	0.000	−0.017 (4)	0.000
C5	0.068 (3)	0.059 (3)	0.093 (4)	0.000	0.015 (3)	0.000
C6	0.061 (2)	0.041 (2)	0.074 (3)	0.000	0.000 (2)	0.000
C7	0.048 (2)	0.076 (4)	0.092 (4)	0.000	−0.011 (3)	0.000
C8	0.058 (3)	0.071 (3)	0.111 (4)	0.000	−0.012 (3)	0.000
C9	0.156	0.156	0.156	0.000	0.000	0.000
C10	0.132 (6)	0.175 (8)	0.136 (6)	0.000	−0.015 (5)	0.000

### Geometric parameters (Å, º)

**Table:**

Cl—C2	1.725 (4)	C3—H3A	0.9300
N1—O1	1.211 (6)	C4—C5	1.360 (7)
N1—O2	1.215 (6)	C4—H4A	0.9300
N1—C5	1.436 (6)	C5—C6	1.415 (6)
C1—C2	1.340 (6)	C7—C8	1.530 (6)
C1—C6	1.403 (6)	C8—C9	1.479 (5)
C1—H1A	0.9300	C8—C9^i^	1.479 (5)
N2—C7	1.363 (6)	C8—C10	1.540 (8)
N2—C6	1.381 (5)	C9—H9A	0.9600
N2—H2A	0.8600	C9—H9B	0.9600
C2—C3	1.396 (7)	C9—H9C	0.9600
O3—C7	1.194 (5)	C10—H10A	0.9600
C3—C4	1.365 (7)	C10—H10B	0.9600
			
O1—N1—O2	119.3 (5)	N2—C6—C1	123.3 (4)
O1—N1—C5	118.4 (6)	N2—C6—C5	121.6 (4)
O2—N1—C5	122.3 (5)	C1—C6—C5	115.1 (4)
C2—C1—C6	122.7 (4)	O3—C7—N2	123.8 (4)
C2—C1—H1A	118.6	O3—C7—C8	119.3 (5)
C6—C1—H1A	118.6	N2—C7—C8	116.9 (4)
C7—N2—C6	128.1 (4)	C9—C8—C9^i^	116.1 (5)
C7—N2—H2A	115.9	C9—C8—C7	110.8 (3)
C6—N2—H2A	115.9	C9^i^—C8—C7	110.8 (3)
C1—C2—C3	121.6 (4)	C9—C8—C10	104.3 (4)
C1—C2—Cl	119.5 (4)	C9^i^—C8—C10	104.3 (4)
C3—C2—Cl	118.9 (4)	C7—C8—C10	109.9 (5)
C4—C3—C2	116.6 (5)	C8—C9—H9A	109.5
C4—C3—H3A	121.7	C8—C9—H9B	109.5
C2—C3—H3A	121.7	H9A—C9—H9B	109.5
C5—C4—C3	123.0 (5)	C8—C9—H9C	109.5
C5—C4—H4A	118.5	H9A—C9—H9C	109.5
C3—C4—H4A	118.5	H9B—C9—H9C	109.5
C4—C5—C6	120.9 (4)	C8—C10—H10A	110.7
C4—C5—N1	117.3 (5)	C8—C10—H10B	108.8
C6—C5—N1	121.8 (5)	H10A—C10—H10B	109.5
			
C6—C1—C2—C3	0.000 (1)	C2—C1—C6—C5	0.000 (1)
C6—C1—C2—Cl	180.0	C4—C5—C6—N2	180.0
C1—C2—C3—C4	0.000 (1)	N1—C5—C6—N2	0.000 (1)
Cl—C2—C3—C4	180.0	C4—C5—C6—C1	0.0
C2—C3—C4—C5	0.000 (1)	N1—C5—C6—C1	180.0
C3—C4—C5—C6	0.000 (1)	C6—N2—C7—O3	0.000 (2)
C3—C4—C5—N1	180.000 (1)	C6—N2—C7—C8	180.000 (1)
O1—N1—C5—C4	0.000 (1)	O3—C7—C8—C9	−114.8 (4)
O2—N1—C5—C4	180.000 (1)	N2—C7—C8—C9	65.2 (4)
O1—N1—C5—C6	180.0	O3—C7—C8—C9^i^	114.8 (4)
O2—N1—C5—C6	0.000 (1)	N2—C7—C8—C9^i^	−65.2 (4)
C7—N2—C6—C1	0.000 (1)	O3—C7—C8—C10	0.000 (2)
C7—N2—C6—C5	180.000 (1)	N2—C7—C8—C10	180.000 (2)
C2—C1—C6—N2	180.0		

Symmetry code: (i) *x*, −*y*+1/2, *z*.

### Hydrogen-bond geometry (Å, º)

**Table:**

*D*—H···*A*	*D*—H	H···*A*	*D*···*A*	*D*—H···*A*
C10—H10*A*···O3^ii^	0.96	2.35	3.294 (8)	167

Symmetry code: (ii) *x*+1/2, −*y*+1/2, −*z*+3/2.
